# Health and kinship matter: Learning about direct-to-consumer genetic testing user experiences via online discussions

**DOI:** 10.1371/journal.pone.0238644

**Published:** 2020-09-08

**Authors:** Zhijun Yin, Lijun Song, Ellen W. Clayton, Bradley A. Malin

**Affiliations:** 1 Department of Biomedical Informatics, Vanderbilt University Medical Center, Nashville, Tennessee, United States of America; 2 Department of Electrical Engineering and Computer Science, Vanderbilt University, Nashville, Tennessee, United States of America; 3 Center for Genetic Privacy & Identity in Community Settings, Vanderbilt University Medical Center, Nashville, Tennessee, United States of America; 4 Department of Sociology, Vanderbilt University, Nashville, Tennessee, United States of America; 5 Center for Biomedical Ethics and Society, Vanderbilt University Medical Center, Nashville, Tennessee, United States of America; 6 Department of Pediatrics, Vanderbilt University Medical Center, Nashville, Tennessee, United States of America; 7 Department of Biostatistics, Vanderbilt University Medical Center, Nashville, Tennessee, United States of America; Independent Research Consultant, UNITED KINGDOM

## Abstract

**Background:**

Millions of people have undergone direct-to-consumer genetic testing (DTC-GT), but little is known about individuals' motivations and experiences (e.g., discussion topics and emotions after obtaining the test results) in engaging with DTC-GT services. Previous studies either involved only a small number of DTC-GT consumers or were based on hypothetical scenarios.

**Objective:**

Our study aimed to fill this gap by investigating online discussions about DTC-GT that developed naturally among tens of thousands of social media users.

**Methods:**

We focused on the posts that were published in the r/23andme and r/AncestryDNA subreddits, which correspond to the two companies with the largest number of consumers in the DTC-GT market. We applied computational methods to infer and examine the topics discussed, temporal trends in posting rates and themes (e.g., aggregation of related topics), and emotions expressed in these online forums.

**Results:**

We collected 157,000 posts published by 16,500 Reddit users between 2013 and 2019. We found that the posting rates increased sharply after popular promotional events (e.g., each Amazon Prime Day and Black Friday) and most posts were inquiries into, or status updates about, testing progress. The inferred themes of Ancestral Origin and Kinship/Feelings were the two most frequently discussed, while discussions about the Health Risks theme focused primarily on submitting DTC-GT raw data to third parties for interpretation. The Kinship/Feelings theme exhibited the largest range of emotional response. A qualitative review of the posts with extreme emotions showed that some people became excited because they found their biological parents or other kin, while others became upset because they unexpectedly found that their parents or other kin were not biologically related to them.

**Conclusion:**

This research demonstrates that online social media platforms can serve as a rich resource for characterizing actual DTC-GT experiences. The findings suggest that DTC-GT consumers' purchasing behaviors are associated with societal events and that future investigations should consider how DTC-GT challenges our understanding of kinship structure and function, genomic privacy, and the interpretation of health risks.

## Introduction

Traditionally, genetic tests have been ordered and interpreted in clinical or biomedical research settings. However, the past decade has witnessed tremendous growth in direct-to-consumer genetic testing (DTC-GT) [1], from which individuals can learn more about themselves regarding a variety of issues, ranging from ascertaining one’s ancestry to assessing the risk of developing various diseases [2, 3]. This type of service is now a commodity offered by an ever-growing collection of companies, the two largest of which are AncestryDNA and 23andMe [4], that has been purchased by more than 25 million people so far [5].

AncestryDNA and 23andMe, as well as many other companies, offer to define from what part(s) of the world one's ancestors originated. Even though the results of such tests can vary across platforms [6, 7], this information has allowed people to uncover membership of a particular tribe or community [2], as well as to understand the relationships among racial and ethnic identities and genetic ancestry in a region [8]. These companies also allow consumers to download their uninterpreted or "raw" sequence data. For example, more than one million people have posted their identified gene data on sites such as GEDMatch in order to find more relatives. The sites where people have posted their DTC-GT sequence results have enabled law enforcement to track down suspects in criminal cases, such as the Golden State Killer [9]. These services also pose non-trivial limitations and consequences for individuals, such as revealing unanticipated information about one’s familial relationships: either identifying new connections or undermining existing ones [3, 10]. Moreover, the same revelations that make it possible to identify relatives and criminal suspects necessarily disclose genomic information about tens of millions of Americans—even if they never underwent DTC genetic testing or consented to sharing information about themselves—creating concerns about privacy intrusion [11].

For years, companies have sought to provide health-related information services, such as genetic risk predispositions to consumers, in conjunction with ancestry and kinship data. This enterprise, however, has been volatile, as illustrated by the numerous companies that have been established and gone defunct [12] and how the U.S. Food and Drug Administration (FDA) famously stepped in to halt 23andMe's health-related business for a time [13]. Yet the availability of DTC health-related products has generated significant concerns by professionals. While some commentators and clinicians reportedly feel comfortable assisting their patients to interpret these results [14, 15], others worry that healthcare providers are ill-prepared or unwilling to assist test recipients who seek advice and medical interventions [16–20] and that these results could increase health care utilization and divert resources from more pressing issues [21–23]. Indeed, several studies indicate that some consumers do seek medical advice but they are not always satisfied [24, 25]. Other consumers plan to engage in self-directed behavioral change but do not always follow through [26, 27].

What is known about consumers' responses to DTC-GT in general, and in regard to health-related results in particular, is incomplete. In a recent systemic literature review of over 150 studies that assessed multiple aspects of these services [28], only nine considered the experience of individuals who actually purchased DTC-GT services [29]. The insights provided by research to date are further limited because they are based on surveys and interviews with consumers [26, 30–33], which reveals responses that may be shaped by the investigators’ questions as well as hindsight bias rather than eliciting the respondents’ unprompted reports of their experiences [34].

Online social platforms provide an opportunity to learn what aspects of DTC-GT are of interest to consumers by examining what they choose to say either on their own or in conversation with others. Indeed, many people use online environments to discuss and share various aspects of their daily lives, including their DTC-GT results. For instance, Twitter users are posting DTC-GT results, typically revealing ancestral background more than other findings (e.g., disease risks) [35]. More recently, a large-scale analysis of Twitter discourse related to DTC-GT indicated that this behavior is often influenced by the news and DTC promotional websites [36]. While many Twitter users disclose their test results, the limited number of characters in a tweet, along with Twitter's design as an all-purpose discussion environment, make it challenging for users to engage in in-depth discussions. Consequently, this medium hinders our ability to gain a deeper understanding of individuals’ questions about, as well as experience with, DTC-GT.

Thus, in this study, we investigated online discussions about taking DTC-GT experience on Reddit, an online content rating and discussion website that was used by approximately 11% of adults in the U.S. in 2019 [37]. Unlike Twitter, which maintains its content based on a social network, Reddit organizes its content into different forums, called subreddits based on particular topics (e.g., r/legaladvice) and allows for posts to be as long as the authors wish. In each subreddit, users can initiate a new thread by submitting a post, make a comment, or upvote another submission or (or downvote) comment. Due in part to its rich content and user engagement, Reddit has grown in popularity for researchers studying a broad range of health issues, including but not limited to mental health, eating disorders, weight loss, dermatological issues, and opioid abuse [38–42].

For this analysis, we specifically focused on the information contributed to the r/23andme and r/AncestryDNA subreddits. We aimed to characterize what people experienced, discussed, and cared about regarding DTC-GT through naturally unfolding online discussions. Particularly, we examined how these topics changed over time, correlated with contemporaneous events such as holiday promotional events, the FDA's approval for 23andMe to provide consumers with health risk reports, and the inferred emotional state of users. We found that many people purchased DTC-GT when there were promotional events, that results regarding ancestry and kinship elicited the most conversation and affect, and that people sought suggestions on sending raw genetic data to third-party services for health risk interpretation. Our findings suggest that some users appeared not to be ready to deal with unexpected consequences from these tests. To the best of our knowledge, this is the first study examining the impact of DTC-GT using long-form consumer-directed online discussions, and our observations reveal the value that this method adds to prior research in DTC-GT area.

## Materials and methods

### Data collection

We collected data from the r/23andme and r/AncestryDNA subreddits through the official Reddit Application Programming Interface. We selected these two subreddits because they provide a specific environment that is dedicated to discussions on the services provided by the two DTC-GT companies that cover the majority of consumers in the market. To collect the data, we created a web crawler using the Python programming language (version 3.6) and applied it to obtain all of the unique identifiers (IDs) of submissions that were archived in pushshift.io before March 26, 2019. Based on these IDs, we next applied the PRAW Python software package (version 5.6.0) to collect submissions as well as any updates, along with comments about them. Each post (which could be a submission or a comment) contained the following fields: 1) post ID, 2) author name, 3) creation date, 4) title (if it is a submission), 5) body text, and 6) the post ID to which it replied.

After collecting the data corpus, we combined the text in the title and body of each submission to represent its content. This was done for two reasons. First, the titles provide information on the topic of the post that is supplemental to the body. Second, we observed that some submissions contained only links in the body of the post. We subsequently removed the posts that contained only a [*delete*] or [*remove*] in the content, which indicated that the post had been deleted by either the authors or mediators of Reddit.

### Topic extraction

We applied latent Dirichlet allocation (LDA) [43], a computational topic modeling technique that is often utilized in natural language processing, implemented in Mallet (version 2.0.8), to identify the general topics that were communicated in r/23andme and r/AncestryDNA subreddits. Given a predefined number of topics (a required parameter for learning LDA) and a large number of posts, LDA generates two distributions. The first corresponds to the probability that each word is used in a topic. The second corresponds to the probability that each topic is used to describe a post. Since LDA is an unsupervised technique (in that it is not trained on examples of known topics), we relied on the coherence score, as well as heuristics based on visualization of the topics, to determine the number of representative topics [44, 45]. The coherence score measures the extent to which two terms in a topic appear together with a high probability in either 1) external documents (e.g., Wikipedia) or 2) the documents that are applied for topic modeling. In general, a larger average coherence score across the topics suggests a better model. At the same time, to enhance the interpretability of topics, we aimed for a lower amount of overlap in topics after their projection into a two-dimensional space according to a multidimensional scaling method. This was accomplished by executing the LDA algorithm with the number of topics equal to each integer between 2 and 25. After each execution, we projected all of the resulting topics into a two-dimensional space and performed a manual review to select the number of topics with a large coherence score and minimal overlap. To further enhance interpretability, we replaced each word with its lemma form and retained only nouns, verbs, adjectives, and adverbs using Spacy (version 2.0.18). It should be noted that we relied on this process to determine whether a word should be retained in the LDA algorithm.

### Topic prevalence

We defined topic prevalence as the percentage of posts that discussed a topic within a fixed time period. Specifically, we followed three steps to calculate prevalence. First, after obtaining the topic distribution for each post, we empirically selected a topic probability threshold to ensure that only the topics with a probability above the threshold are acknowledged as being present in a given post. For instance, imagine that we set a threshold of 0.25 in a ten-topic model and a post exhibits probability of 0.3 for topic T_1_, 0.3 for topic T_2_, and 0.05 for each of the remaining eight topics. In this situation, we say that only topics T_1_ and T_2_ are present in this post. Second, once the threshold was determined, we created a topic co-occurrence matrix by computing when two topics were present as the primary (with the highest probability) and the secondary (with the second highest probability) topics in a post. The value of each matrix cell represents the percentage of posts that discussed the corresponding primary and/or secondary topics. Third, we aggregated the topics into more general themes based on topic co-occurrence. The identified themes were then subject to a prevalence analysis.

### Emotions in the themes

After generating themes, which indicated what DTC-GT users experienced, we further compared the emotions in different themes to obtain greater insight into how they felt about their testing experience. Specifically, we applied Linguistic Inquiry Word Count (LIWC, version 2015) to extract the percentage of words in a post that mentions either a positive or negative emotion [46]. LIWC has been widely applied to user-generated data in online environments, including Twitter and Reddit, to perform semantic investigations into discussions about various health- and wellness-related issues such as post-traumatic stress disorder [47], and characterize conversational patterns [48]. We applied a Wilcoxon signed-rank test and Mann–Whitney U test to compare the rate at which the emotions were expressed across the themes at a significance level of 0.001.

## Results

### Data summary

We collected 19,744 posts (2,403 submissions; 17,341 comments) published by 2,562 Reddit users between June 30, 2017 and March 26, 2019 in r/AncestryDNA, and 138,008 posts (13,543 submissions; 124,465 comments) published by 14,983 Reddit users between December 31, 2012 and March 26, 2019 in r/23andme. It should be recognized that r/AncestryDNA was initiated in 2017 while r/23andme was initiated four years earlier in 2013. This is intuitive because 23andMe started its service in 2006 while AncestryDNA ran its first test in 2012. However, it is still surprising that the number of Reddit users in r/AncestryDNA (~2,500) was substantially smaller than that in r/23andme (~15,000) because this is the opposite of their market penetration. According to 2019 estimates, AncestryDNA had approximately 5 million more users than 23andMe [5]. Given that the number of users differed, we inquired if there was a difference in the rate at which Reddit users participated in these subreddits. It was observed that, on average, each user in r/AncestryDNA wrote 7.7 posts compared to 9.2 posts in r/23andme. Yet this difference was not statistically significant under a Wilcoxon rank-sum test (p = 0.07), which suggested that the rate of contribution did not influence the following analysis.

### Posting trends

**Fig 1:**
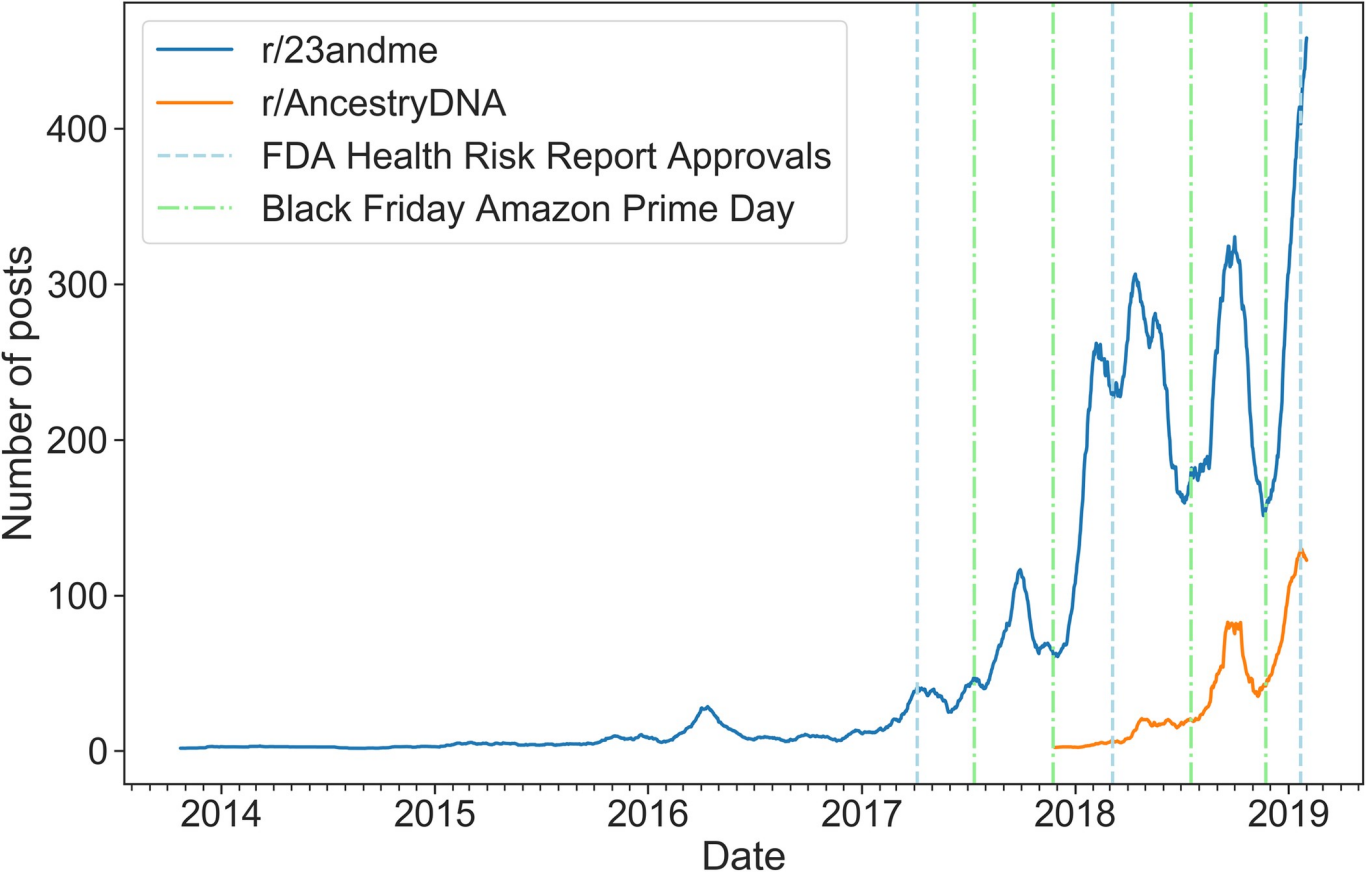
A 30-day moving average of the number of posts in r/AncestryDNA and r/23andme since 2017.

Fig 1 illustrates the temporal posting trends in r/AncestryDNA and r/23andme. The Black Fridays and Amazon Prime Days in 2017 and 2018, as well as the three dates when the FDA granted 23andMe approval to sell products associated with health risk assessments (April 2017; March 2018; and January 2019), are indicated by vertical dashed lines. Overall, the posting rates increased in both subreddits over time. It should be noted that by May 8, 2019, r/23andme already had 29,878 subscribers and ranked 4,802 in popularity among more than 1 million subreddits. Moreover, the posting rates exhibited strong periodic patterns. After each Black Friday and Amazon Prime Day, the posting rates escalated rapidly in both subreddits. The posting rate also grew markedly after the second FDA approval date (March 6, 2018), and the growth in the posting rate after Black Friday in 2018 continued after the date of the third FDA approval (January 22, 2019).

### Post topics

Table 1 summarizes the nine topics inferred from r/AncestryDNA and r/23andme through LDA (see S1 Fig for the visualization of the nine topics). The rightmost column reports the topic distribution when all the posts are combined into a single document. The summary labels were assigned by the authors based on an examination of the relevant terms and posts. There are several notable findings to highlight.

**Table 1:** Topics inferred from a joint analysis of the r/AncestryDNA and r/23andme subreddits.

Label	Most Relevant Terms^a^	Distribution
**Kinship**	family, dad, father, mom, parent, cousin, great, side, half, mother, match, share, find, tree, grandfather, sister, grandmother, sibling, child, brother	0.119
**Health Risks**	test, genetic, datum, gene, information, health, give, good, company, andme, testing, research, promethease, work, read, issue, problem, run, raw, service	0.115
**DNA Matching**	dna, ancestry, andme, show, relative, country, ancestor, grandparent, report, match, percentage, test, share, population, customer, change, bear, accurate, base, recent	0.115
**Feelings**	make, find, good, thing, feel, time, give, happen, hear, question, talk, story, life, answer, contact, idea, work, hope, wrong, guy	0.112
**European Ancestry**	european, german, italian, irish, french, ancestry, british, europe, broadly, jewish, ashkenazi, eastern, southern, region, scandinavian, high, english, iberian, ancestor, expect	0.112
**General Ancestry**	american, native, asian, african, white, south, east, european, north, people, group, spanish, chinese, mexican, part, black, mix, west, mixed, middle_eastern	0.111
**Haplogroup**	people, lot, back, pretty, year, haplogroup, common, ago, interesting, live, family, cool, history, line, guess, true, make, generation, lol, bit	0.110
**Sharing Results**	result, https, andme, imgur, post, reddit, update, gedmatch, www, guess, phase, link, comment, upload, site, ancestrydna, myheritage, http, test, add	0.104
**Testing Progress**	day, kit, receive, extraction, week, sample, time, wait, mine, send, analysis, long, report, today, email, update, check, generation, step, process	0.103

^a^The terms were ranked according to their probability in the distribution per topic. The labels were assigned by the authors as a readable summary of the topic.

First, Kinship and Health Risks were the two most discussed topics in terms of the number of words. Within the topic of Health Risks, users mainly sought information about third-party interpretation to learn about their disease risk. The following are several representative posts on these two topics (note that these quotes are not meant to be definitive proof and should be viewed as exemplars only):

“*Located my biological dad*. *This is my son and my father at the same age*. *I used ancestryDNA and other tools to find him […]” (Kinship topic probability*: *0*.*196)*

“*Today*, *I ran the raw data through Promethease and Codegen*. *They show a ton of increased risks*, *for almost everything […]” (Health Risks topic probability*: *0*.*233)*

Second, there were two topics related to ancestral origin: European Ancestry and General Ancestry (e.g., American, African, Asian, and Mexican), and another two topics related to Haplogroup and DNA Matching. The following are two representative posts:

“*Surprising number of distant relatives share same segment of DNA on 8*^*th*^ *chromosome–Anomaly*? *Any Explanations*? *[…]” (DNA Matching topic probability*: *0*.*349)*

“*Where does this haplogroup come from*, *where are you from*? *American native Americans are what haplogroup*?*” (Haplogroup topic probability*: *0*.*153)*

Third, many users of these two subreddits disclosed their feelings. A post from one user demonstrates this topic with respect to their search experience for kin:

“*I found my biological family*! *[…] I don’t feel much of a thing but a little anxiety […] this experience has made me feel so much more grateful for my [current] family […] wish I had never sent my test back*.*” (Feelings topic probability*: *0*.*301)*

Finally, people discussed their Testing Progress, disclosed uploading or sharing testing results activities in either online websites or third-party services:

“*AncestryDNA data uploaded to WeGene*, *gencove*, *DNA*.*Land*, *GEDmatch*, *and MyHeritageDNA […]” (Sharing Results topic probability*: *0*.*196)*

### Topic similarity between subreddits

Table 2 provides a comparison of the distributions of topics in r/23andme after the second FDA approval and r/AncestryDNA. First, it was found that users in r/23andme were more likely to talk about the Testing Progress and Health Risks topics, while users in r/AncestryDNA were more likely to talk about Kinship. Second, when talking about their ancestry, users in r/23andme were more likely to mention heritage in general, while users in r/AncestryDNA were more likely to mention European heritage. Additionally, users in r/23andme were more likely to mention Sharing Results, Feelings, and Haplogroup topics.

**Table 2:** The difference in topic probabilities between r/23andme and r/AncestryDNA (after April 2017).

Topic	r/23andme	r/AncestryDNA	Difference^a^
**Testing Progress**	0.1136	0.1025	0.0110
**Health Risks**	0.1101	0.1051	0.0050
**General Ancestry**	0.1121	0.1105	0.0016
**Sharing Results**	0.1098	0.1084	0.0014
**Feelings**	0.1116	0.1105	0.0011
**Haplogroup**	0.1102	0.1094	0.0009
**DNA Matching**	0.1102	0.1106	-0.0004
**Kinship**	0.1122	0.1206	-0.0084
**European Ancestry**	0.1102	0.1224	-0.0122

^a^The difference for each topic was statistically significant on a Mann-Whiney U test at the <0.001 confidence level.

### Topic prevalence

We generated the topic co-occurrence matrix (see S2 Fig) by empirically setting the distribution threshold to 0.13 (i.e., topics with a probability below 0.13 were deemed to be insufficiently representative of a post). We empirically selected this threshold based on a requirement that it should be larger than the average topic distribution (e.g. 0.11). Based on the matrix, we clustered the topics into six themes: 1) Testing Progress; 2) Ancestral Origin (European Ancestry, General Ancestry); 3) Sharing Results; 4) Health Risks; 5) Haplogroup/Matching (Haplogroup, DNA Matching); and 6) Kinship/Feelings.

**Fig 2:**
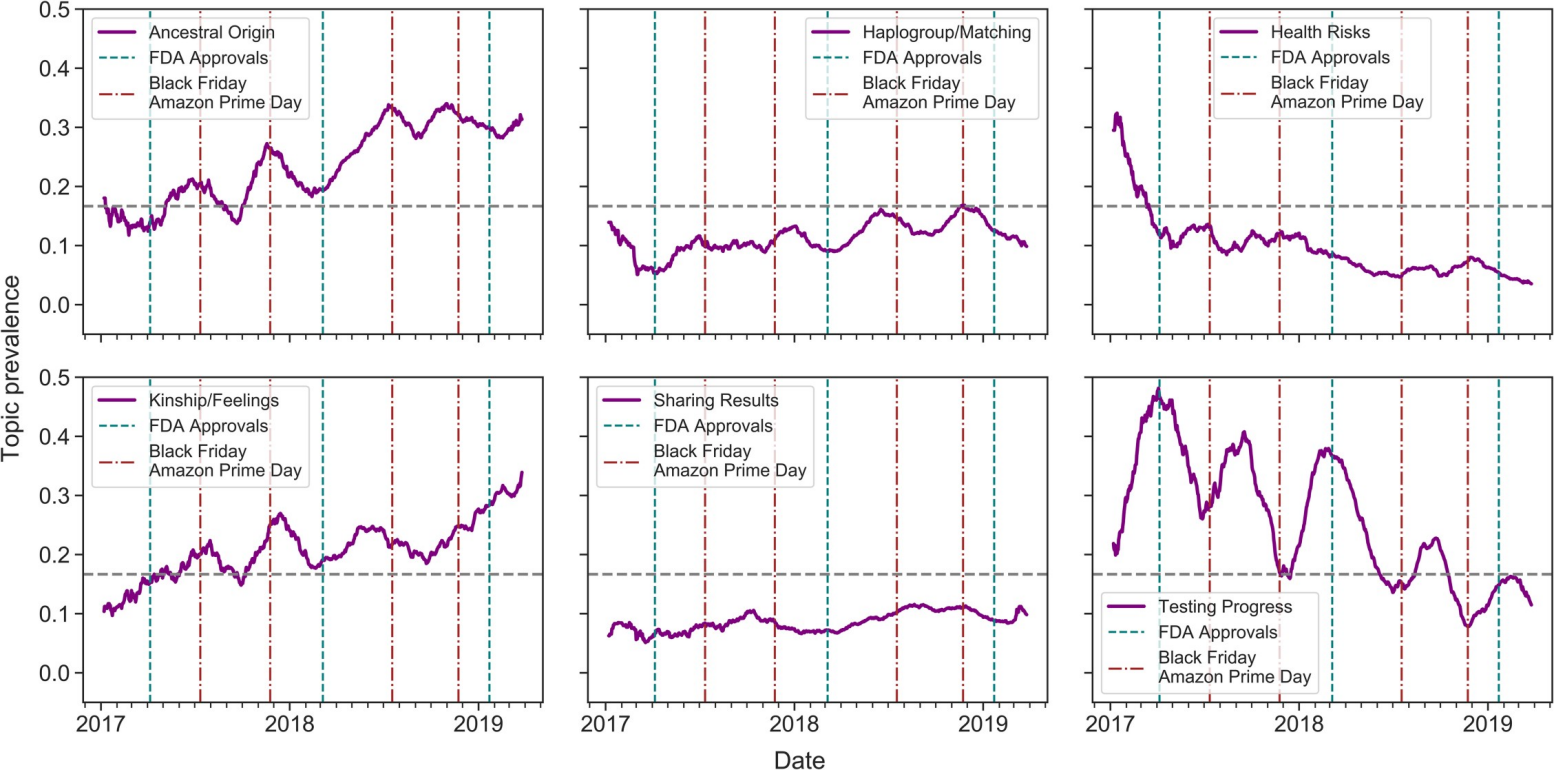
A 90-day moving average of the number of posts communicating the inferred themes. The horizontal dashed line corresponds to the average topic prevalence (1/6 = 0.167). "FDA Approvals" represent the dates when the FDA granted 23andMe authorization for DTC health risk reports.

Fig 2 illustrates the temporal prevalence of the six themes. In this analysis, we combined both subreddits due to the relatively small number of posts in r/AncestryDNA. There are several findings worth highlighting. First, we observed that the Ancestral Origin and Kinship/Feelings themes increased in prevalence over time, eventually achieving levels that were substantially higher than the average prevalence. Second, the Haplogroup/Matching, Health Risks, and Sharing Results themes were below the average level most of the time. Third, the Testing Progress theme decreased in prevalence, but experienced periodicity that was highly correlated with Black Friday and Amazon Prime Day in 2017 and 2018, as previously noted.

### Emotions in the themes

Table 3 compares the two inferred emotions between the themes. The (P)ositive and (N)egative columns show the average percentage of the corresponding emotion words identified by applying LIWC for every post in each theme. ΔN and ΔP refer to a comparison between each theme with the Kinship/Feelings for both emotions, respectively. P-N refers to the difference between the two emotions for each theme. Here, there are several notable observations. First, the subreddit users expressed positive emotions more often than negative emotions across all the themes (as shown in the P-N column). Second, the Kinship/Feelings exhibited the highest negative emotion, which was followed by the Health Risks and Testing Progress themes (as shown in the ΔN column). Third, the Kinship/Feelings theme also exhibited the highest positive emotion, followed by the Haplogroup/Matching and Health Risks themes (as shown in the ΔP column).

**Table 3:** A comparison of emotions across themes.

Theme	(N)egative	(P)ositive	ΔN^a^	ΔP^a^	P-N^b^
**Kinship/Feelings**	0.0199	0.0530	-	-	0.0332
**Health Risks**	0.0133	0.0355	0.0066	0.0176	0.0222
**Testing Progress**	0.0120	0.0225	0.0079	0.0305	0.0106
**Genealogy/Matching**	0.0080	0.0462	0.0119	0.0069	0.0382
**Sharing Results**	0.0062	0.0355	0.0137	0.0175	0.0294
**Ancestral Origin**	0.0054	0.0234	0.0145	0.0297	0.0180

^a^ΔN and ΔP refer to a comparison of each theme with the Kinship/Feelings based on the Mann-Whitney U test (P < 0.001), respectively.

^b^P-N is a comparison of the positive and negative emotions based on the Wilcoxon signed-rank test (P < 0.001).

## Discussion

### Principal findings

This investigation of online discussion about DTC-GT in two subreddits yielded several primary findings. First, the topics discussed by the Reddit users align with the services offered by the DTC-GT companies. Of particular note, r/AncestryDNA users were more likely to discuss European ancestry composition as compared with the world more generally, which is likely due to the fact that 296 of its 392 (75.5%) ethnic regions are for people of European heritage, while only 52 of 171 (30.4%) in 23andMe are European [49], differences which themselves are striking.

Second, the observed posting trends in both subreddits clearly reflect the impact of consumer marketing. For example, both DTC-GT and the number of posts published in the subreddits have experienced rapid growth since 2017 [50]. Additionally, it was evident that Reddit users’ purchasing behaviors were associated with major promotional events, as illustrated by marked spikes in activity after each Black Friday and Amazon Prime Day, when both companies achieved strong holiday sales on Amazon.com due to price reductions [51]. As one user mentioned in a post:

*“[…] I order mine on Black Friday on Amazon for a total of $106*. *And got my results in January. It was the ancestry+health […]”*

By contrast, approvals by the FDA appeared to have little impact on purchases of tests from 23andme.

Third, Ancestral Origin and Kinship/Feelings were the two most frequently discussed themes in these two subreddits, but kinship was a more prominent topic and was accompanied by a wider array of emotions. Some people sought to find new relatives, which is consistent with the growing body of literature that focuses particularly on people who were adopted [52] or who were conceived using gamete donation [53]. That interest in uncovering these relationships is driven in part by a desire for information about health history [54] or even identity is particularly intriguing since, until recently, the norm in the law in this country had been severing these connections [55]. On other occasions, people discovered that their biological connections were not what they expected, an occurrence increasingly documented in the media. For example, a recent cover story published by the American Psychological Association reported that a woman who planned to seek testing with her fiancé before marriage unexpectedly discovered that she had been conceived with donor sperm [56]. A similar story was reported in r/23andme as well:

*“Ordered myself and my dad a kit when they were on sale […] the first thing […] was to compare ancestry composition […] a little bit off*. *[…] then went to DNA relatives […] didn’t pop up on mine […] dad got so angry […] My mom […] admitted I was some other man’s child […] they are now divorcing […]”*

Indeed, some writers assert that family secrets may be a thing of the past [57]. Yet, DTC companies say little about these potential revelations, at most including language in their terms of service that people may be surprised by the results.

Fourth, discussions about health risks were less common but focused primarily on submitting DTC-GT generated raw data to third parties for interpretation. Given the level of concern among health care providers about DTC health-related results [22, 24, 58] and the many studies of individuals' views about these tests [59, 60], the level of spontaneous discussion of the test results offered by 23andMe was surprisingly low even after 2017 when that company was once again permitted to offer them. This may be attributable to the limited panel of health-related results offered by 23andMe, most of which are relatively uncommon, so that most discussants would not have received a concerning result. Being found to be a carrier of a recessive disorder would be more common, but likely to be less distressing unless the person was actively pursuing childbearing [61]. And of course, some of those who did receive worrisome results may not have chosen to disclose them in online environments.

Many people, however, clearly wanted health-related information, as evidenced by the fact that most of the posts regarding health focused on the possibility of using third-party services to interpret health risks from raw DTC-GC data, which had often been obtained from purchasing a basic ancestry service. This is a path pursued by a growing number of consumers [62]. Yet using third-party services to obtain health information is problematic, even if clinicians are willing to consider them. Third-party interpretation websites often lack adequate informed consent, have questionable clinical validity and utility, and lack medical supervision [63]. Moreover, one study reported that 40% of genetic variations identified from DTC-GT raw data were not confirmed on further analysis in a clinical laboratory [3]. Thus, consumers may receive inaccurate results.

### Limitations and future work

Despite the insights gained from this study, there are several limitations that we believe can serve as the basis of future work. First, the population in our study was composed of active users in r/23andme and r/AncestryDNA, which may limit the generalizability of our findings. For example, Reddit users are typically male (69%), between the ages of 18 and 29 (64%), Caucasian (70%), from the United States (58%), and completed at least some college education [64], which differs from the demographics of 23andme and Ancestry users who tend to be older and more often female but are still mostly Caucasian and college educated [65, 66]. It could be useful to consider users from other online platforms, and other strategies to understand the experiences of non-social-media users to obtain broader insights. Second, we relied on LDA to discover the topics and focused on providing a general picture of discussion in these subreddits. Future investigations can apply alternative advanced topic modeling techniques, such as structural topic models, to directly extract topic prevalence [67]. Third, while LIWC is a linguistic tool designed to support semantic analysis in social media data, there are other tools dedicated to emotion analysis, such as the NRC-lexicon [68] and the EMOTIVE-ontology [69], that could be utilized. Fourth, it would be worthwhile to investigate the extent to which online discussion helps individuals cope with the consequences of undergoing DTC-GT. Finally, it will also be important to monitor whether discussions about using DTC-GT to learn health risks grow as the FDA approves more of these tests, particularly those that purport to assess common disease risk, and as other companies begin to offer them [70, 71].

## Conclusion

This investigation presented evidence that online social media platforms can serve as a rich resource for characterizing actual DTC-GT experiences, yielding insights that can complement research strategies that rely on elicited responses to surveys and interviews. In particular, for DTC-GT consumers who disclosed their experience in r/23andme and r/AncestryDNA, we observed that their discussion focused on kinship, with both positive and negative consequences, inquiries or updates on testing progress, ancestral origin, and intent to send raw DNA data to third parties for health risk interpretations. The findings suggest that DTC-GT consumer’s purchase behaviors are associated with societal events (e.g., holiday promotions) and that future investigations will need to consider how DTC-GT challenges notions of kinship structure and function, genomic privacy, as well as health risk interpretation.

## Supporting information

S1 Fig Topic visualization. The topic index in each circle is corresponding to the presenting order of the topics in Table 1.
(TIF)

S2 Fig The rate at which topics co-occur in the subreddits. Each cell represents the percentage of posts that mentioned the corresponding combination of topics. Cells along the top-left to bottom-right diagonal correspond to posts that expressed one topic only. The matrix was generated by empirically setting the distribution threshold as 0.13 (i.e., topics with probability below 0.13 were deemed to be insufficiently representative of a post).
(TIF)

## References

[1] Khan R, Mittelman D. Consumer genomics will change your life, whether you get tested or not. Genome Biol. 2018;19(1): 120 10.1186/s13059-018-1506-1 30124172 PMC6100720

[2] Walajahi H, Wilson DR, Hull SC. Constructing identities: the implications of DTC ancestry testing for tribal communities. Genet Med. 2019;21(8): 1744–1750. 10.1038/s41436-018-0429-2 30662065 PMC6642857

[3] Tandy-Connor S, Guiltinan J, Krempely K, LaDuca H, Reineke P, Gutierrez S, et al False-positive results released by direct-to-consumer genetic tests highlight the importance of clinical confirmation testing for appropriate patient care. Genet Med. 2018;20: 1515–1521. 10.1038/gim.2018.38 29565420 PMC6301953

[4] Hazel J, Slobogin C. Who knows what, and when?: a survey of the privacy policies proffered by us direct-to-consumer genetic testing companies. Cornell J Law Public Policy. 2018;28(1): 35–66. 30840416

[5] Regalado A. Rewriting life more than 26 million people have taken an at-home ancestry test. 2019 [cited 20 Apr 2019]. Available from: https://www.technologyreview.com/s/612880/more-than-26-million-people-have-taken-an-at-home-ancestry-test/

[6] Phillips AM. Only a click away—DTC genetics for ancestry, health, love…and more: A view of the business and regulatory landscape. Appl Transl Genom. 2016: 8: 16–22. 10.1016/j.atg.2016.01.001 27047755 PMC4796702

[7] Garrison NA. Genetic ancestry testing with tribes: ethics, identity & health implications. Daedalus. Journal of the American Academy of Arts & Sciences. 2018;147(2): 60–69. 10.1162/DAED_a_00490

[8] Bryc K, Durand EY, Macpherson JM, Reich D, Mountain JL. The genetic ancestry of african americans, latinos, and european Americans across the United States. Am J Hum Genet. 2015;96(1): 37–53. 10.1016/j.ajhg.2014.11.010 25529636 PMC4289685

[9] Erlich Y, Shor T, Pe’er I, Carmi S. Identity inference of genomic data using long-range familial searches. Science. 2018;362: 690–694. 10.1126/science.aau4832 30309907 PMC7549546

[10] Crawshaw M. Direct-to-consumer DNA testing: the fallout for individuals and their families unexpectedly learning of their donor conception origins. Hum Fertil. 2018;21(4): 225–228. 10.1080/14647273.2017.1339127 28697325

[11] Ducharme J. Millions of Americans could be identified using consumer genetic databases—even if they’ve never taken a DNA test. 2018 [cited 20 Apr 2019]. Available from https://time.com/5423170/dna-test-identify-millions/.

[12] Allyse MA, Robinson DH, Ferber MJ, Sharp RR. Direct-to-consumer testing 2.0: emerging models of direct-to-consumer genetic testing. Mayo Clinic Proceedings. 2018; 93(1):113–120. 10.1016/j.mayocp.2017.11.001 29304915

[13] Check Hayden E. The rise and fall and rise again of 23andMe. Nature. 2017;550(7675): 174–177. 10.1038/550174a 29022933

[14] Brothers KB, Knapp EE. How should primary care physicians respond to direct-to-consumer genetic test results? AMA Journal of Ethics. 2018;20: 812–818. 10.1001/amajethics.2018.812 30242811

[15] Bernhardt BA, Zayac C, Gordon ES, Wawak L, Pyeritz RE, Gollust SE. Incorporating direct-to-consumer genomic information into patient care: attitudes and experiences of primary care physicians. J Pers Med. 2012;9(7): 683–692. 10.2217/pme.12.80 23795206 PMC3684987

[16] Storrs C. Patients armed with their own genetic data raise tough questions. Health Aff. 2018;37(5): 690–693. 10.1377/hlthaff.2018.0364 29733706

[17] Carroll JC, Makuwaza T, Manca DP, Sopcak N, Permaul JA, O’Brien MA, et al Primary care providers’ experiences with and perceptions of personalized genomic medicine. Can Fam Physician. 2016;62: e626–e635. 27737998 PMC5063789

[18] Powell KP, Cogswell WA, Christianson CA, Dave G, Verma A, Eubanks S, et al Primary care physicians’ awareness, experience and opinions of direct-to-consumer genetic testing. J Genet Couns. 2012;21(1): 113–126. 10.1007/s10897-011-9390-9 21769569

[19] McBride CM, Alford SH, Reid RJ, Larson EB, Baxevanis AD, Brody LC. Characteristics of users of online personalized genomic risk assessments: Implications for physician-patient interactions. Genet Med. 2009;11(8): 582–587. 10.1097/GIM.0b013e3181b22c3a 19606049 PMC3341609

[20] Rockwell KL. Direct-to-consumer medical testing in the era of value-based care. J Am Med Assoc. 2017;317(24): 2485–2486. 10.1001/jama.2017.5929 28542699

[21] Pet DB, Holm IA, Williams JL, Myers MF, Novak LL, Brothers KB, et al Physicians’ perspectives on receiving unsolicited genomic results. Genet Med. 2019; 21(2): 311–318. 10.1038/s41436-018-0047-z 29904163 PMC6294706

[22] Bloss CS, Schork NJ, Topol EJ. Direct-to-consumer pharmacogenomic testing is associated with increased physician utilisation. J Med Genet. 2014;51(2): 83–89. 10.1136/jmedgenet-2013-101909 24343916

[23] Giovanni MA, Fickie MR, Lehmann LS, Green RC, Meckley LM, Veenstra D, et al Health-care referrals from direct-to-consumer genetic testing. Genet Test Mol Biomarkers. 2010;14(6): 817–819. 10.1089/gtmb.2010.0051 20979566 PMC3001829

[24] Van Der Wouden CH, Carere DA, Maitland-Van Der Zee AH, Ruffin MT, Roberts JS, Green RC, et al Consumer perceptions of interactions with primary care providers after direct-to-consumer personal genomic testing. Ann Intern Med. 2016; 164(8): 513–522. 10.7326/M15-0995 26928821

[25] Hollands GJ, French DP, Griffin SJ, Prevost AT, Sutton S, King S, et al The impact of communicating genetic risks of disease on riskreducing health behaviour: Systematic review with meta-analysis. BMJ. 2016;352: i1102 10.1136/bmj.i1102 26979548 PMC4793156

[26] Carere DA, VanderWeele TJ, Vassy JL, others. Prescription medication changes following direct-to-consumer personal genomic testing: findings from the impact of Personal Genomics (PGen) study. Genet Med. 2017;19: 537–545. 10.1038/gim.2016.141 27657683 PMC5362351

[27] Kaufman DJ, Bollinger JM, Dvoskin RL, Scott JA. Risky business: Risk perception and the use of medical services among customers of DTC personal genetic testing. J Genet Couns. 2012;21(3):413–422. 10.1007/s10897-012-9483-0 22278220

[28] Salloum RG, George TJ, Silver N, others. Rural-urban and racial-ethnic differences in awareness of direct-to-consumer genetic testing. BMC Public Health. 2018;18: 277 10.1186/s12889-018-5190-6 29471813 PMC5824539

[29] Covolo L, Rubinelli S, Ceretti E, Gelatti U. Internet-based direct-to-consumer genetic testing: a systematic review. J Med Internet Res. 2015;17: e279 10.2196/jmir.4378 26677835 PMC4704942

[30] Francke U, Dijamco C, Kiefer AK, Eriksson N, Moiseff B, Tung JY, et al Dealing with the unexpected: consumer responses to direct-access BRCA mutation testing. J Life Environ Sci. 2013;1: e8 10.7717/peerj.8 23638402 PMC3628894

[31] Motzer RJ, Escudier B, Gannon A, Figlin RA. Sunitinib: Ten years of successful clinical use and study in advanced renal cell carcinoma. Oncologist. 2017;22: 41–52. 10.1634/theoncologist.2016-0197 27807302 PMC5313263

[32] Bloss CS, Schork NJ, Topol EJ. Effect of direct-to-consumer genomewide profiling to assess disease risk. N Engl J Med. 2011;364: 524–534. 10.1056/NEJMoa1011893 21226570 PMC3786730

[33] Hartz SM, Olfson E, Culverhouse R, Cavazos-Rehg P, Chen LS, DuBois J, et al Return of individual genetic results in a high-risk sample: enthusiasm and positive behavioral change. Genet Med. 2015;17: 374–9. 10.1038/gim.2014.110 25166427 PMC4344933

[34] Yin Z, Malin B, Warner J, Hsueh P-Y, Chen C-H. The power of the patient voice: learning indicators of treatment adherence from an online breast cancer forum. Proceedings of the Eleventh International AAAI Conference on Web and Social Media 2017;2017:337–346.

[35] Olejnik L, Agnieszka K, Castelluccia C. I’M 2.8% Neanderthal-the beginning of genetic exhibitionism? Workshop on Genome Privacy. 2014 Available from http://seclab.soic.indiana.edu/GenomePrivacy/papers/Genome%20Privacy-paper4.pdf

[36] Mittos A, Blackburn J, De Cristofaro E. “23andMe confirms: I’m super white”—analyzing twitter discourse on genetic testing. arXiv preprint arXiv:180109946. 2018.

[37] Perrin A, ANDERSON M. Share of U.S. adults using social media, including Facebook, is mostly unchanged since 2018. 2019 [cited 20 Apr 2019]. Available from: https://www.pewresearch.org/fact-tank/2019/04/10/share-of-u-s-adults-using-social-media-including-facebook-is-mostly-unchanged-since-2018/

[38] Park A, Conway M. Harnessing reddit to understand the written-communication challenges experienced by individuals with mental health disorders: analysis of texts from mental health communities. J Med Internet Res. 2018;20(4): e121 10.2196/jmir.8219 29636316 PMC5915669

[39] Buntinx-Krieg T, Caravaglio J, Domozych R, Dellavalle RP. Dermatology on reddit: elucidating trends in dermatologic communications on the world wide web. Dermatol Online J. 2017;23(7):13030/qt9dr1f7x6. 29469693

[40] Sowles SJ, McLeary M, Optican A, Cahn E, Krauss MJ, Fitzsimmons-Craft EE, et al A content analysis of an online pro-eating disorder community on Reddit. Body Image. 2018;24: 137–144. 10.1016/j.bodyim.2018.01.001 29414146 PMC5869127

[41] D’Agostino AR, Optican AR, Sowles SJ, Krauss MJ, Escobar Lee K, Cavazos-Rehg PA. Social networking online to recover from opioid use disorder: a study of community interactions. Drug Alcohol Depend. 2017;181: 5–10. 10.1016/j.drugalcdep.2017.09.010 29024875 PMC5683917

[42] Liu Y, Yin Z. Understanding weight loss via online discussions: content analysis of Reddit posts using topic modeling and word clustering techniques. J Med Internet Res. 2020; 22(6):e13745 10.2196/13745 32510460 PMC7308899

[43] Blei DM, Edu BB, Ng AY, Edu AS, Jordan MI, Edu JB. Latent dirichlet allocation. J Mach Learn Res. 2003;3: 993–1022. 10.1162/jmlr.2003.3.4–5.993

[44] Röder M, Both A, Hinneburg A. Exploring the space of topic coherence measures. Proceedings of the eighth ACM international conference on Web search and data mining. 2015;2015: 399–408. 10.1145/2684822.2685324

[45] Sievert C, Shirley K. LDAvis: A method for visualizing and interpreting topics. Proceedings of the Workshop on Interactive Language Learning, Visualization, and Interfaces. 2014;2014: 63–70. doi: 10.1.1.100.1089

[46] Chung CK, Pennebaker JW. Linguistic inquiry and word count (LIWC). Applied Natural Language Processing. 2012:2012: 206–229. 10.4018/978-1-60960-741-8.ch012

[47] Coppersmith G, Harman C, Dredze M. Measuring post traumatic stress disorder in twitter. Proceedings of the 8th International Conference on Weblogs and Social Media. 2014;2014: 579–582.

[48] Choi D, Han J, Chung T, Ahn YY, Chun BG, Kwon T. Characterizing conversation patterns in reddit: From the perspectives of content properties and user participation behaviors. Proceedings of the 2015 ACM Conference on Online Social Networks. 2015;2015: 233–243. 10.1145/2817946.2817959

[49] Holger D. 23andMe vs. AncestryDNA: what’s the difference? 2018 [cited 20 Apr 2019]. Available from: https://www.pcworld.com/article/3322523/23andme-vs-ancestry-dna.html

[50] Regalado A. 2017 was the year consumer DNA testing blew up. 2018 [cited 20 Apr 2020]. Available from https://www.technologyreview.com/2018/02/12/145676/2017-was-the-year-consumer-dna-testing-blew-up/.

[51] Estrada M. 23andMe’s DNA Test was a best-seller on Black Friday, and it’s discounted again for Cyber Monday. 2017 [cited 20 Apr 2020]. Available from https://www.yahoo.com/news/23andme-dna-test-best-seller-black-friday-discounted-130653190.html.

[52] Lee H, Vogel RI, LeRoy B, Zierhut HA. Adult adoptees and their use of direct‐to‐consumer genetic testing: searching for family, searching for health. J Genet Couns.in press. 10.1002/jgc4.1304 32602181

[53] Forshall G. Hertz R. and Nelson M.K. Random families: genetic strangers, sperm donor siblings and the creation of new kin, New York: Oxford University Press; 2019 10.1111/1467-9566.13033

[54] May T, Strong KA, Zusevics KL, Jeruzal J, Farrell MH, LaPean Kirschner A, et al Does lack of “genetic-relative family health history” represent a potentially avoidable health disparity for adoptees? Am J Bioeth. 2016;16: 33–38. 10.1080/15265161.2016.1240255 27901440

[55] Jacobson H. Anonymity in third party reproduction: an old dilemma in new packaging? J Law Biosci. 2016;3: 660–665. 10.1093/jlb/lsw035 28852545 PMC5570695

[56] Pappas S. Genetic testing and family secrets. 2018 [cited 12 Aug 2020]. Available from: https://www.apa.org/monitor/2018/06/cover-genetic-testing

[57] There’s no such thing as family secrets in the age of 23andme. 2020 [cited 12 Aug 2020]. Available: https://www.wired.com/story/theres-no-such-thing-as-family-secrets-in-the-age-of-23andme/

[58] Hsieh V, Braid T, Gordon E, Hercher L. Direct-to-consumer genetic testing companies tell their customers to ‘see a genetic counselor’. How do genetic counselors feel about direct-to-consumer genetic testing? Genet Couns. in press. 10.1002/jgc4.1310 32706156

[59] Savard J, Hickerton C, Metcalfe SA, Gaff C, Middleton A, Newson AJ. From expectations to experiences: consumer autonomy and choice in personal genomic testing. AJOB Empir Bioeth. 2020; 11(1): 63–76. 10.1080/23294515.2019.1701583 31885332 PMC7048070

[60] Ruhl GL, Hazel JW, Clayton EW, Malin BA. Public attitudes toward direct to consumer genetic testing. AMIA Annu Symp Proc. 2020;2019: 774–783. 32308873 PMC7153088

[61] Ramos E, Weissman SM. The dawn of consumer-directed testing. Am J Med Genet Semin Med Genet. 2018 3;178(1):89–97. 10.1002/ajmg.c.31603 29512889

[62] Wang C, Cahill TJ, Parlato A, Wertz B, Zhong Q, Cunningham TN, et al Consumer use and response to online third-party raw DNA interpretation services. Mol Genet Genomic Med. 2018;6(1):35–43. 10.1002/mgg3.340 29471590 PMC5823680

[63] Badalato L, Kalokairinou L, Borry P. Third party interpretation of raw genetic data: an ethical exploration. Eur J Hum Genet. 2017;25(11): 1189–1194. 10.1038/ejhg.2017.126 28832567 PMC5643961

[64] Sattelberg W. The demographics of Reddit: who uses the site? 2020 [cited 12 Aug 2020] Available from: https://social.techjunkie.com/demographics-reddit/.

[65] Shelton JF, Cameron B, Aslibekyan S, 23andMe Research Team, Gentleman R. Demographic, spatial and temporal dietary intake patterns among 526 774 23andMe research participants. Public Health Nutr. 2020; 1–12. 10.1017/S1368980020001251 32597744 PMC9884798

[66] Horowitz AL, Saperstein A, Little J, Maiers M, Hollenbach JA. Consumer (dis-)interest in genetic ancestry testing: the roles of race, immigration, and ancestral certainty. New Genet Soc. 2019; 38(2): 165–194. 10.1080/14636778.2018.1562327 31814797 PMC6897494

[67] Roberts ME, Stewart BM, Tingley D. Stm: An R package for structural topic models. J. Stat. Softw. 2019;91(2): 1–40. 10.18637/jss.v091.i02

[68] Mohammad SM, Turney PD. Crowdsourcing a word-emotion association lexicon. Comput Intell-US. 2013;29(3): 436–465. 10.1111/j.1467-8640.2012.00460.x

[69] Sykora MD, Jackson TW, O’Brien A, Elayan S. Emotive ontology: extracting fine-grained emotions from terse, informal messages. Proceedings of the IADIS International Conference Intelligent Systems and Agents. 2013;8(2): 106–118

[70] Ancestry begins genetic testing services for health. 2019 [cited 12 Aug 2020]. Available from: https://www.360dx.com/business-news/ancestry-begins-genetic-testing-services-health

[71] MyHeritage launches health-related genetic gest, ignites debates. 2019 [cited 12 Aug 2020]. Available from: https://www.the-scientist.com/news-opinion/myheritage-launches-health-related-genetic-test—ignites-debate-66115

