# Supplementary figures and images for: Health and kinship matter: Learning about direct-to-consumer genetic testing user experiences via online discussions
Source: PLoS One. 2020 Sep 8;15(9):e0238644. doi: 10.1371/journal.pone.0238644 (PMC7478842; doi:10.1371/journal.pone.0238644)

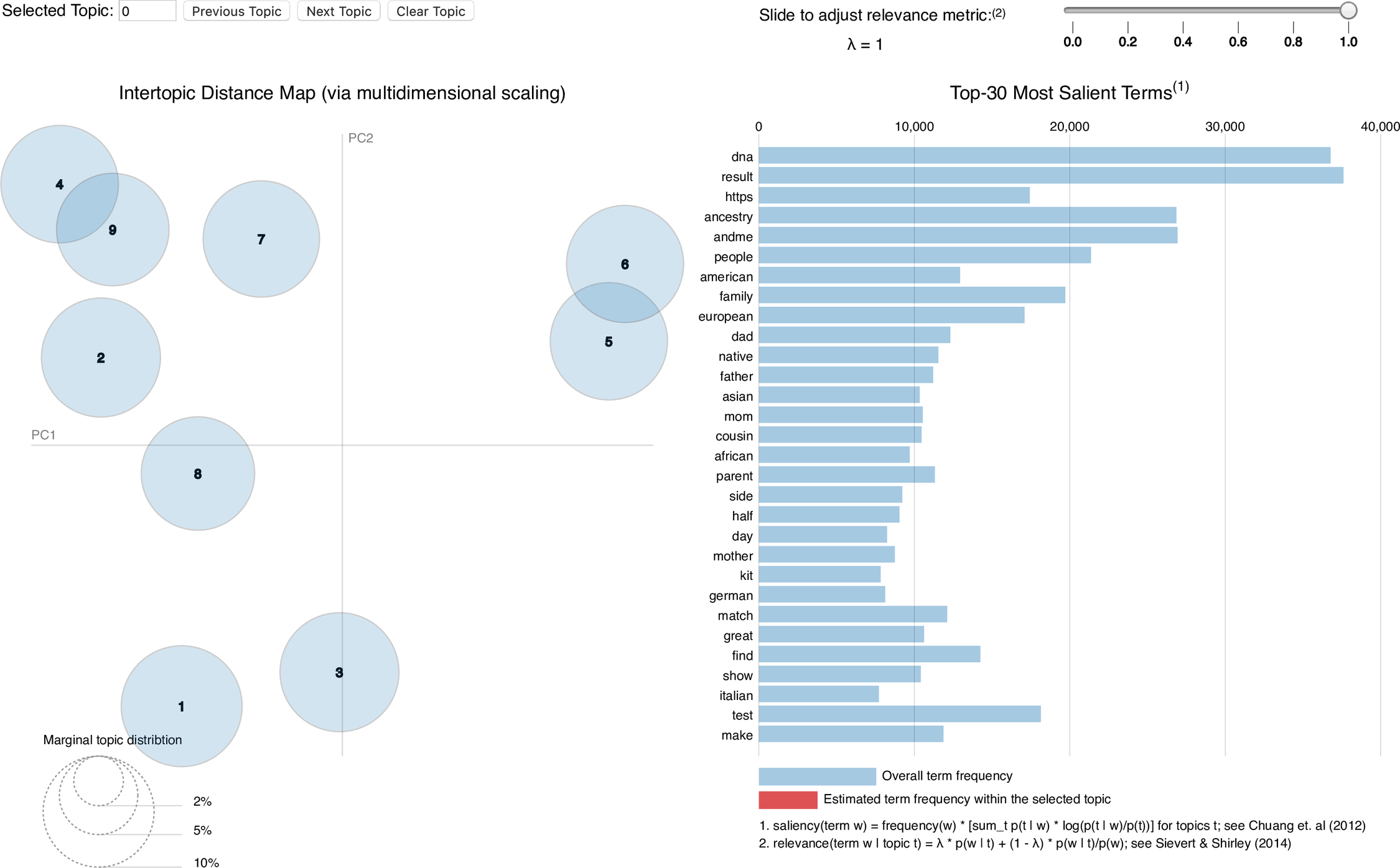

Supplement: S1 Fig — The topic index in each circle is corresponding to the presenting order of the topics in Table 1. (TIF) [file pone.0238644.s001.tif]

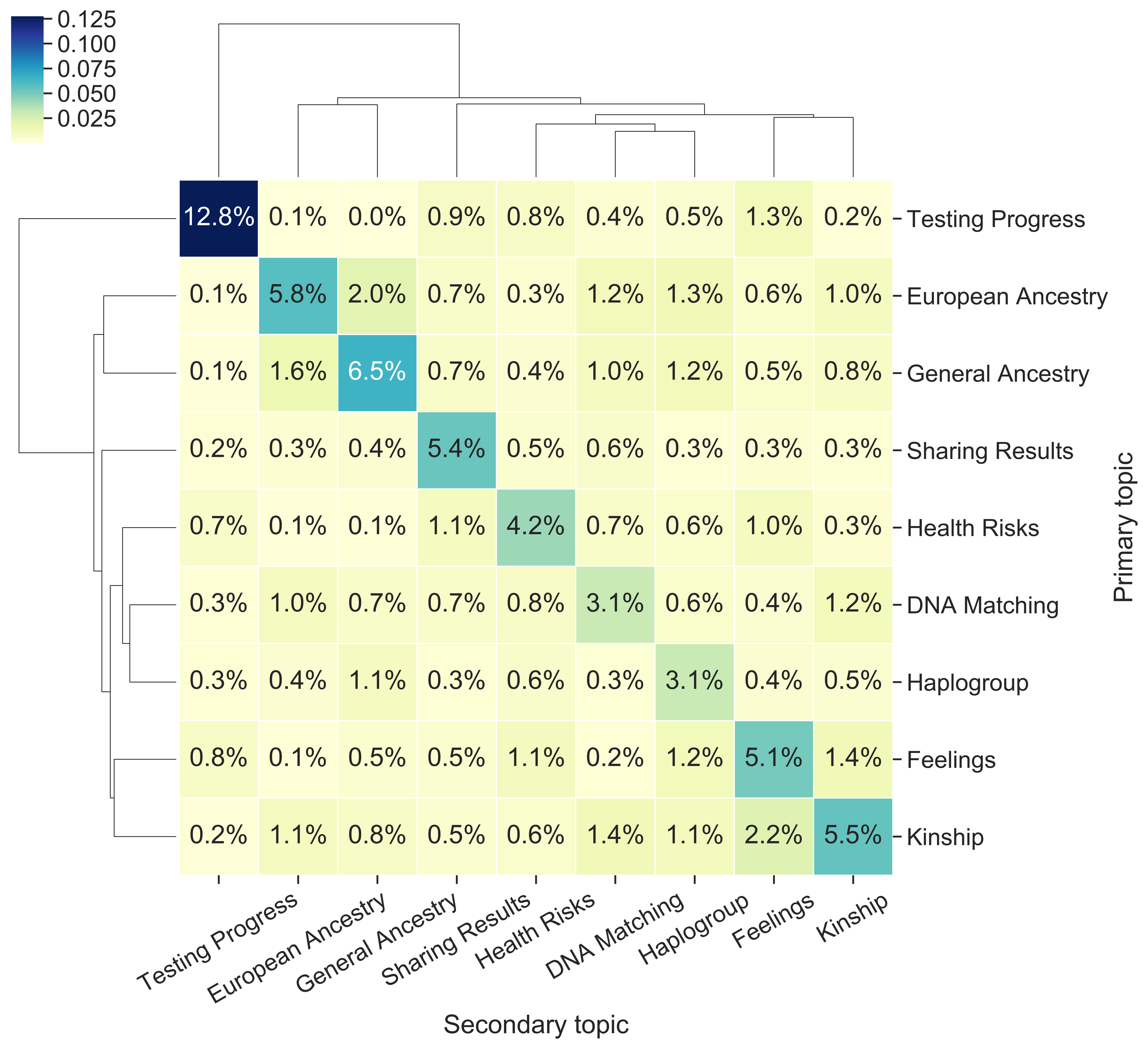

Supplement: S2 Fig — Each cell represents the percentage of posts that mentioned the corresponding combination of topics. Cells along the top-left to bottom-right diagonal correspond to posts that expressed one topic only. The matrix was generated by empirically setting the distribution threshold as 0.13 (i.e., topics with probability below 0.13 were deemed to be insufficiently representative of a post). (TIF) [file pone.0238644.s002.tif]
